# One Health, Fermented Foods, and Gut Microbiota

**DOI:** 10.3390/foods7120195

**Published:** 2018-12-03

**Authors:** Victoria Bell, Jorge Ferrão, Lígia Pimentel, Manuela Pintado, Tito Fernandes

**Affiliations:** 1 Faculdade de Farmácia, Universidade de Coimbra, Azinhaga de Santa Comba, 3000-548 Coimbra, Portugal; victoriabell1103@gmail.com; 2 Universidade Pedagógica, Rua João Carlos Raposo Beirão 135, Maputo 1000-001, Mozambique; ljferrao@icloud.com; 3 CBQF-Centro de Biotecnologia e Química Fina, Escola Superior de Biotecnologia, Universidade Católica Portuguesa, Rua Arquiteto Lobão Vital, Apartado 2511, 4202-401 Porto, Portugal; lpimentel@porto.ucp.pt (L.P.); mpintado@porto.ucp.pt (M.P.); 4 Faculdade de Medicina Veterinária, Universidade de Lisboa, 1300-477 Lisboa, Portugal

**Keywords:** nutrition, probiotics, fermented foods, health benefits

## Abstract

Changes in present-day society such as diets with more sugar, salt, and saturated fat, bad habits and unhealthy lifestyles contribute to the likelihood of the involvement of the microbiota in inflammatory diseases, which contribute to global epidemics of obesity, depression, and mental health concerns. The microbiota is presently one of the hottest areas of scientific and medical research, and exerts a marked influence on the host during homeostasis and disease. Fermented foods and beverages are generally defined as products made by microbial organisms and enzymatic conversions of major and minor food components. Further to the commonly-recognized effects of nutrition on the digestive health (e.g., dysbiosis) and well-being, there is now strong evidence for the impact of fermented foods and beverages (e.g., yoghurt, pickles, bread, kefir, beers, wines, mead), produced or preserved by the action of microorganisms, on general health, namely their significance on the gut microbiota balance and brain functionality. Fermented products require microorganisms, i.e., *Saccharomyces* yeasts and lactic acid bacteria, yielding alcohol and lactic acid. Ingestion of vibrant probiotics, especially those contained in fermented foods, is found to cause significant positive improvements in balancing intestinal permeability and barrier function. Our guts control and deal with every aspect of our health. How we digest our food and even the food sensitivities we have is linked with our mood, behavior, energy, weight, food cravings, hormone balance, immunity, and overall wellness. We highlight some impacts in this domain and debate calls for the convergence of interdisciplinary research fields from the United Nations’ initiative. Worldwide human and animal medicine are practiced separately; veterinary science and animal health are generally neither considered nor inserted within national or international Health discussions. The absence of a clear definition and subsequent vision for the future of One Health may act as a barrier to transdisciplinary collaboration. The point of this mini review is to highlight the role of fermented foods and beverages on gut microbiota and debate if the need for confluence of transdisciplinary fields of One Health is feasible and achievable, since they are managed by separate sectors with limited communication.

## 1. Introduction

The microbiota exerts a marked influence on the host during homeostasis linked with metabolic diseases in humans, but demonstration of causality remains a challenge [1]. 

Humans as hosts have co-evolved with microorganisms over millions of years, and each body habitat has a unique set of microorganisms shaping its microbiota [2].

These bacteria live on the skin, in the corners of the eyes, in the oral cavity, under fingernails, and most importantly, in the guts. Several perinatal determinants, such as caesarean section delivery, type of feeding, the use of antibiotics, gestational age or environment can affect the pattern of bacterial colonization, resulting in gut dysbiosis. The establishment and development of the gut microbiota over the lifecycle moved from the previous accepted dogma that the mammalian healthy placenta and foetus were germ-free and considered to be sterile, and that these conditions were critical to the developing newborn’s immune system, to the actual knowledge that in utero humans are now known to harbour unique prenatal microbiomes [3,4].

Amniotic fluid may contain microorganisms, increasing the complexity of fetal microbiota, and having implications for the long-term health and susceptibility to disease, as placental microbiota could trigger immune responses in the fetus. Early gut microbiota settlement influences the maturation of the infant’s immune system [5] and subsequent health, although the evidence in support of the “in utero colonization hypothesis” is considered extremely weak by some authors [6].

Health authorities are now becoming fully aware that one cannot be considered to be in good health without a well-balanced microbiota composition in the gut, our “forgotten organ” [7], and of the fundamental role of a diverse and healthy gut microbiota on the subsequent maintenance of future health and well-being of the host [8,9]. Indeed, although it is broadly mentioned that there are 10 times more cells from microorganisms in our bodies than there are human cells [10], this claim has been challenged, and others have estimated that the number of bacteria is similar to that of human cells [11]. 

Many species of bacteria, specifically those found in the invisible universe of the human microbiota, e.g., composed of nonpathogenic commensal microbiota from the *Firmicutes*, *Bacteroidetes*, *Actinobacteria*, *Proteobacteria* and *Verrucomicrobia* phyla [12], are unsusceptible to petri dish cultivation. They can be successfully cultivated in association with other microbes, meaning in communities of different bacteria species. But without being able to isolate them, research is difficult [13]. Commensal microbiota gradually deteriorates in sick patients. Therefore, research is being conducted to generate new technologies to study the rest of the human microbiome using advances in DNA-sequencing technologies and associated computational methods [14]. Metagenomic sequencing of total fecal DNA samples offers complementary support to classical microbiology, and enables researchers to access previously-inaccessible genomic information from gut bacteria [15,16]. 

In recent years, a number of functional species and strains have been identified in human metabolic diseases [17]. Gut bacteria can produce various bioactive metabolites which can be detrimental to the host’s health, such as those with cytotoxicity, genotoxicity, or immunotoxicity [18], shifting the paradigm of understanding the root cause of the onset and progression of several human metabolic diseases [19].

Gut microbiota modulates the expression of many genes in the human intestinal tract [20], including genes involved in immunity, nutrient absorption, energy metabolism, and intestinal barrier function. It is important to understand genomic diversity of specific members of the gut microbiota if precise nutrition-based approaches are to be realized [21]. 

In the oral use of live bacteria, there is more research concerning isolated probiotic commercial supplements than there is work concerning health benefits of common fermented foods, since major industries usually do not fund this type of research [22]. Many studies suggest that probiotics may help with diarrhea or symptoms of irritable bowel syndrome, but strong evidence to support their use for most health conditions is lacking in people with sepsis, and probiotics are no panacea [23,24]. 

Probiotics should not be universally given as a ‘one-size-fits-all’; most trials were based on stool samples, which may not really reflect the bacteria living in the gut, as shedding takes place continuously [25]. Besides, taking probiotics after treatment with broad spectrum antibiotics may actually delay the return of normal gut microbiome, a new potential adverse side effect [26]. 

The One Health concept, introduced at the beginning of the 2000s [27], is a worldwide strategy for promoting multidisciplinary partnerships and information in all facets of health care sciences, perceiving the interrelationship between humans, animals, plants, and their common environment [28]. By working with physicians, veterinarians, osteopathic physicians, dentists, pharmacists, nurses, ecologists, wildlife professionals, and other scientific-health and environmentally-related specialists, it will be possible to monitor and control public health threats and learn how diseases spread among people, animals, and the environment [29]. 

**Figure 1:**
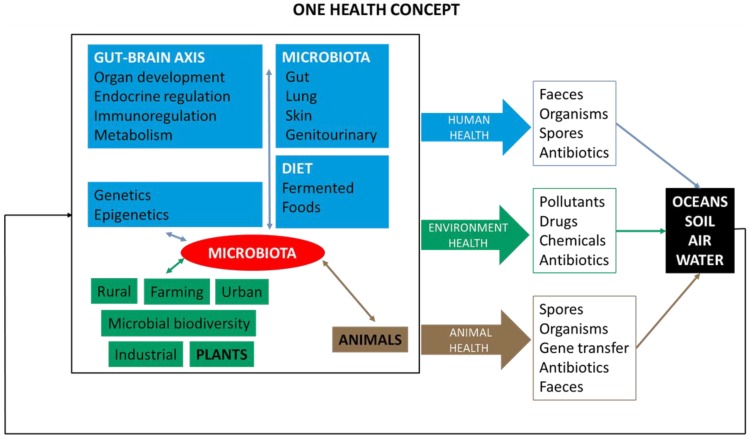
A general picture of the One Health (Human-Animal-Environment) concept as a trans-disciplinary effort. Contribution of the three branches of public health. Microbiota and human metabolic diseases, animal health, and environmental epidemiology.

The point of this mini review is to highlight if the requirement for multiconvergence of the research fields of One Health (Human-Animal-Environment), the relationship between microbiota-nutrition and fermented foods, and to underline the idea that future gut-brain research is feasible and achievable (Figure 1).

## 2. Microbiota and General Health

Having an active and natural variety of microorganisms in the gut may improve general health [30]. The good, healthy bacteria make food more digestible through their enzymes, increased vitamin synthesis, and the preservation of nutrients, and also help to reduce sweet cravings, maintain the immune system, and benefit overall gut wellness [31]. 

The microbiome, consisting of microorganisms and their collective genomes, modulates the host metabolic phenotype, and influences the host immune system. It is now well established that gut bacteria are closely tied to immune health [32]. The gut microbiota regulates l-tryptophan metabolism and identifies the underlying molecular mechanisms of these interactions [33]. 

A large majority of the immune system resides in the tonsils and gut, so when gut health is imbalanced, it is hard for the body’s immune system to function properly [34]. There are also a number of common factors in modern life that can throw human gut bacteria off, such as processed foods and antibiotics. The use of antibiotics does have several short and long-term implications in the ecology of the normal microbiota and gut motility [35]. 

Research on the health benefits of probiotics is still emerging, mainly from the food and beverage industries and their commercial interests. In contrast, strong, independent scientific evidence to support specific uses of probiotics for most health conditions is still lacking [36]. 

The administration of probiotics/prebiotics has been shown to alter the composition and functionality of the gut microbiota [37]. Recent evidence indicates that the effects of probiotics are likely to be different from one person to the next [38]. 

In addition, probiotics might be ineffective, and possibly counterproductive, in restoring the baseline gut microbiome after it has been altered by antibiotic treatment. Indeed, probiotics may not be quite as good as was commonly thought, and they could even be harmful if taken after antibiotics [39].

Serious disorders such as obesity, anorexia, irritable bowel syndrome, autism, and posttraumatic stress disorder—which have been thought to be solely psychological—share a common symptom: a hypersensitivity to gut stimuli [40,41,42].

The role of environmental factors in the development of autism is a crucial and an important area of research concerning how the environment influences and interacts with genetic susceptibility. Factors such as parental age at conception, maternal nutrition, infection during pregnancy, and premature birth are risk factors [43]. Autism (ASD, autism spectrum disorder), a developmental disorder characterized by disturbance in language, perception, and socialization, with no exact known cause, is usually linked with bioenergetic metabolism deficiency [44] and neuro-inflammatory conditions [45], and immune system dysregulation and dreadful gut concerns may improve with better diet and fermented foods (e.g., fermented raw coconut milk) [46,47]. 

Specific benefits from the direct dietary modulation of the human gut microbiota has been described [48]. Despite the wide array of beneficial mechanisms deployed by probiotic bacteria and fermented foods and beverages, relatively few effects have been supported by clinical data [49]. 

**Figure 2:**
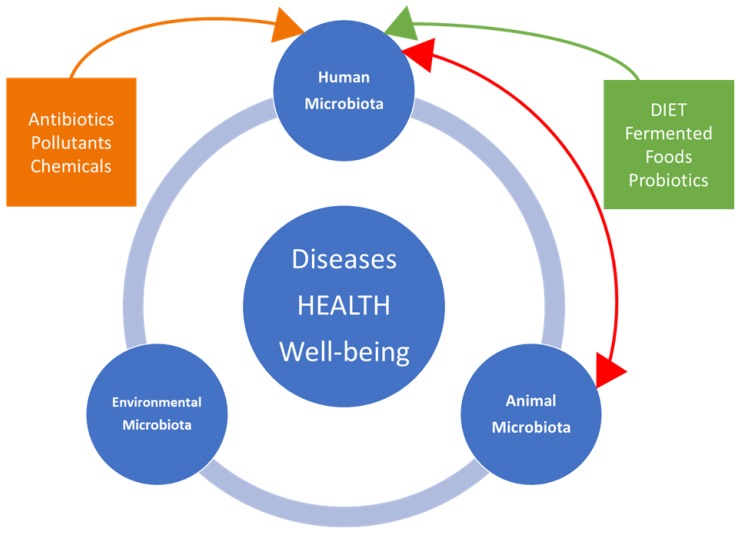
Interactions between dynamics of microbiota in humans, animals, and the associated environment with disease occurrence, salubrity, and well-being.

The interactions (Figure 2) between ingested fermented food and intestinal microbiota, and their correlations to metabolomics profiles and health, represent an important perspective, and independent research on health benefits is still emerging [50,51]. Microbiota is specific to each individual, despite the existence of several bacterial species shared by the majority of adults. A diverse and propitious microbial ecosystem (e.g., *Bacteroides fragilis*, *Bifidobacterium* spp. and *Faecalibacterium* spp.) favors homeostasis, particularly at the level of the disease–immune dialogue [52,53]. 

## 3. Fermented Foods, Probiotics, Body and Mind

The use of fermentation in conserving food and beverage as a means to provide better taste, improve nutrition and food safety, organically preserve foodstuffs, and promote health properties, is a well-known ancient practice. The reasons for fermenting foods and beverages include improvements of a product’s storage time, safety, functionality, organoleptic quality, and nutritional quality properties [54]. Not only is this process beneficial for extending shelf-life, but also, fermentation can enhance nutritional properties in a safe and effective manner [55].

Many types of food groups, including dairy, vegetables, legumes, cereals, starchy roots, and fruits, as well as meat and fish, can be fermented [56]. Fermented foods and beverages can comprise anywhere from 5–40% of the human diet in some populations [57].

Phytochemicals, defined as the non-nutritive, naturally-occurring chemicals found in fruits, vegetables, wholegrains, legumes, beans, herbs, spices, nuts, and seeds, are responsible for producing physiological properties, as well as protecting against various environmental stressors of the plant crops. There are more than one thousand known phytochemicals (e.g., lycopene in tomatoes, isoflavones in soy, and flavanoids in fruits). The microbiota comes into contact with a wide variety of dietary components that escape gut digestion and may be affected by phytochemicals [58]. 

**Figure 3:**
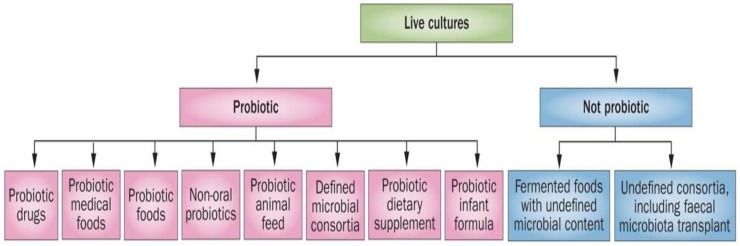
Overall framework for probiotic products [64].

Substantial confusion exists between fermented foods and beverages and the probiotic concept. It is important to address the common misconception that fermented foods are the same thing as probiotics [59]. They are not probiotics, although they may contain them, as their live microbial content is undefined. The term “probiotic” was first coined [60] in 1974, and many authors have described the history and the progress of probiotics and their different applications. Ilya Ilyich Metchnikoff, the Nobel Prize winner in Medicine in 1908, was the first who observed the effect of what is called now “probiotic” [61]. FAO/WHO redefined the term “probiotics”, which is now widely accepted as constituting “live microorganisms that, when administered in adequate amounts, confer a health benefit on the host” [62]. Different types of bacteria (e.g., *Lactobacillus*, *Bifidobacterium*, *Streptococcus*, *Bacillus*) and yeast or mold (e.g., *Saccharomyces*, *Aspergillus*, *Candida*) are used as probiotics. Probably, the first real use of food containing probiotics was fermented milk, but today we have to differentiate between probiotics and probiotic-containing foods (e.g., fermented foods) [63]. The scope and appropriate use of the term “probiotic” has been well clarified (Figure 3) [64].

Probiotics are able to renew, restore, and grow affected tissues lining the digestive tract with beneficial microorganisms neutralizing the harmful ones. Useful live microorganisms will regenerate our microflora fermenting our food correctly and improving our health [65,66].

Despite the impact of fermented foods and beverages on gastro-intestinal well-being and diseases, their health benefits or recommended consumption have not been widely translated to global inclusion in world food guidelines [67]. When fermented foods and beverages are supplemented with probiotic bacteria, they provide numerous extra nutritional and health characteristics [68]. 

Fermented foods and beverages are more popular than ever before, while research into the health benefits of fermented foods is relatively new. Not all fermented foods contain live organisms; beer and wine, for example, undergo steps that remove the organisms, and other fermented foods like bread are heat-treated and the organisms are inactivated. The strain composition and stability of the microbes in fermented foods is not well understood [69].

Fermentation generates adjustments in yeast and live microorganisms cultures in the absence of air, but retains the enzymes, vitamins, and minerals in foods and beverages, which are usually destroyed by processing [70]. The fermenting microorganism, bacteria or yeast, plays a precious role in the functional property of fermented foods and beverages [71]. One the biggest benefits of fermented foods comes from the probiotics they might contain [72]. There are currently no authorized European health claims for probiotics, and the application of probiotics is controversial, since the European Food Safety Authority (EFSA) rejected all submitted health claims related to the term “probiotic”, while accepting the term “live microorganism cultures” in yoghurt [73].

Traditional and modern dietary practices utilize fermented foods and beverages, contributing significantly to the food chain value and belonging to a category of foods called “functional foods” (e.g., probiotics, prebiotics, stanols and sterols) by having an additional characteristic, i.e., health-promotion or disease prevention effect [74].

Fermentation converts sugars, in the absence of oxygen, into organic acids, gases, alcohols, and carbon dioxide, and provides several benefits such as new and desirable tastes and textures, enhancement of nutrients (e.g., linoleic acid; bioactive peptides), removal of toxic or undesirable food constituents (e.g., phytic acid; bitter-tasting phenolic compounds), delivery of probiotic bacteria (e.g., *Lactobacillus delbrueckii* subsp. *bulgaricus*; *Streptococcus thermophilus*), and inhibition of foodborne pathogens [75,76].

Fermented foods and beverages are useful because they help provide a spectrum of probiotics to foster a vigorous microbiome. Fermented foods with unidentified microbial content cannot be considered probiotic suppliers. The two main effects of the daily consumption of fermented foods are upon the immune system and upon metabolic function [77]. 

Dealing with fermented foods has parallels with One Health, since it involves the links between human, animal, environment, foods and microbiota that impacts the organoleptic and physicochemical characteristics of foods as well as human health [78].

There are well documented effects of how adverse early life influences on the gut-brain axis and the use of fermented foods and beverages, mainly with probiotic bacteria, can restore a disturbance of the normal luminal habitat, and so change the effects of the central nervous system on the microbiota [79].

Our guts control and deal with every aspect of our health. How we digest our food, and even the food sensitivities we have are linked to our mood, behavior, energy, weight, food cravings, hormone balance, and immunity [80]. The interaction of nutrients with the microbiota is essentially what determines overall health. Eating and drinking fermented foods and beverages, especially organic unpeeled and unpasteurized fruits and vegetables, improves the bioaccessibility and bioavailability of food bioactive components, supplying dietary fibers and essential micronutrients such as trace-elements and phytochemicals, together with enzymes, lactic acid bacteria, and organic acids, all of which are crucial for good health [81]. 

Changes in the human colonic microbiota fingerprint are associated with the major causes of morbidity and mortality worldwide, diabetes and cardiovascular diseases, due to imbalances between beneficial and pathogenic bacteria [82]. 

Physiologically-active peptides with different functionalities are produced from food proteins during fermentation and food digestion by lactic acid bacteria. In some fermented products, bioactive peptides (e.g., immunoglobulins, antibacterial peptides, antimicrobial proteins, oligosaccharides, lipids, and other “minor” components) have the potential to be used in the formulation of health-enhancing nutraceuticals [83], and include short amino acid sequences that, upon release from the parent protein, may play different physiological roles, including antioxidant, antihypertensive, antimicrobial, and other bioactivities [84,85].

Fermentation may enhance the benefits of a wide variety of foods, dairy products, herbs, and beverages, acting upon the absorption and activity of their secondary metabolites and chemical elements [86]. However, it is not always possible to clearly distinguish the potential contribution of the microbial content from that of the food matrix. There is recent evidence and consumer perception of the health benefits of fermented foods and beverages [87], beyond the popular recognized effects on the impairment of gastrointestinal function, namely, their relevance on gut microbiota, correlated to human health and to several infectious [88], inflammatory, and neoplastic disease processes [89], as well as to brain functionality [90].

Despite disagreement among mental health practitioners and researchers pertaining to the aetiology, categorization, and medical care of several mental disorders [91], current research regarding fermented foods, the microbiome, and their effect on human health, particularly the global epidemic of mental health [92], describes problems associated with the modern lifestyle, and with the western diet being high in sugar and saturated fat [93].

The degradation of the intestinal mucous membrane, weakening the tight barrier against the ingress of harmful substances, and the protection against a reaction to omnipresent harmless compounds, is a primary cause of several disturbances [94]. 

Ingestion of vibrant probiotics, especially in fermented foods, is found to cause significant positive improvements in balancing intestinal permeability and barrier function [95], with direct effects on metabolic syndrome, atherosclerosis, inflammatory bowel diseases, and colon cancer [96] and indirect effects on depression, anger, anxiety, and levels of stress hormones [97].

Young individuals with autism often have a reduced number of microorganisms in the gut [98], and atypical digestive health conditions may occur, like chronic gastrointestinal and functional bowel disorder, causing discomfort, diarrhea and bloating, abdominal pain and cramping, collectively described as irritable bowel syndrome [99]. Children with autism spectrum, besides having a genetic predisposition, show a disruption of the indigenous gut flora and an elevated number of potentially pathogenic (toxin-producing) *Clostridia* in the gut [100,101]. The effectiveness of fermented foods, mushroom biomass, and probiotics in relieving gut symptoms in autistic children has been studied [102,103,104].

The involvement of the microbiota in inflammatory diseases may contribute to altered mood via intestinal permeability, systemic and local lipopolysaccharide burden, and even direct-to-brain microbial communication [105]. In future, insights based upon omics techniques will increase our knowledge between pathogens and healthy strains, thereby explaining food ecosystems and their dynamics [106,107]. 

## 4. Fermented Foods in Developing Settings

Around the world, each culture has its own distinctiveness in terms of food culture and heritage, where fermented foods are included. In the developing world, for people living in poverty, the main priority is not food hygiene, safety, and nutritive factors, as they consume less nutritious foods in which chemical, microbiological, zoonotic, and other hazards may pose a health risk [108].

African traditional fermented foods and beverages have been used since ancient times. Throughout the continent, there is great variety of fermented foods and beverages, mainly sour porridges and drinks. Of the various types of fermentations used to obtain fermented foods and beverages, lactic acid and alcoholic fermentations are the most popular in developing settings, where some 80% of the population still seek care from traditional healers who prescribe indigenous products.

In Africa, a continent which consumes high levels of lactic acid fermented products, estimates for mental disorders and depression vary widely, but seemingly, such diseases are not less common than in developed societies [109], although factors other than diet exist which may exacerbate conditions such as socio-economic changes, urbanicity, alterations in dietary habits, and, more recently, sedentary behavior among youth [110]. 

People from Sub-Saharan Africa, often plagued by civil conflicts, drought, floods, famine, and disease, but with huge biodiversity of plants and herbs, tend to rely on traditional healers who often interpret mental illness in terms of possession or curses, and tackle mental health by rituals, but also by recommending traditional plants, herbs, fermented foods, and beverages [111,112]. Many rural communities in Africa are totally reliant on traditional fermented foods as the primary source of nutrition for nourishment, as well as for cultural traditional practices [113].

In Mozambique and Zimbabwe, traditional fermented foods are used for weaning from the age of four months. The commonest fermented food is known are *mahewu*, a traditional, fermented, malted, sour, non-alcoholic maize or cassava thin porridge, sour milk and sour porridge [114,115]. The Tanzanian fermented gruel, *togwa*, has been found to protect against foodborne illnesses in regions that have poor sanitation [116]. 

However, each person is unique in their needs and sensitivities. Most of us only think of histamine when thinking of allergies. Indigenous fermented foods and beverages, as potential sources of probiotics, may be very therapeutic for some, while others may have an intolerance to histamine since there is no histamine free diet, and this amine, with many functions in the body, occurs naturally, and is a neurotransmitter in the central nervous system [117]. 

Fruits and vegetables are easily perishable commodities in Africa due to their high water activity and nutritive values. This phenomenon is more critical in tropical and subtropical countries, whose climates favor the growth of spoilage causing microorganisms. It is in developing settings in Africa, Asia [118], and Latin America [119,120] that perhaps the greatest need for probiotics and fermented foods exist; however, for many reasons, this is not the case [121]. 

## 5. One Health Approach and International Organizations

Antimicrobial resistance is the ability of a microorganism (like bacteria, viruses, and some parasites) to stop an antimicrobial (such as antibiotics, antivirals, and antimalarials) from working against it. As a result, standard treatments become ineffective, infections persist, and may spread to others.

Antibiotic resistance is one of the biggest threats to global health, food security, and development today, and can affect anyone, of any age, in any part of the world. Bacteria, not humans or animals, become antibiotic-resistant; the cause for this is mainly the way antibiotics are prescribed and used without sales supervision and medical or veterinarian control. Tackling antibiotic resistance is a high priority for the United Nations’ agencies FAO, and OIE, and the WHO, who are leading multiple initiatives and global action plans.

The United Nations (UN) has become the foremost forum in addressing issues that transcend national boundaries and cannot be resolved by any single country acting alone. While conflict resolution and peacekeeping continue to be among its most visible efforts, the UN, along with its specialized agencies, is also engaged in a wide array of activities to improve people’s lives around the world—from disaster relief, through education and advancement of women, to peaceful uses of atomic energy.

Despite great successes since 1953, the UN has, in the past and presently, experienced a number of catastrophic failures, such as the war on sustainable development, global energy goals, refugee and climate change policies, famine, poverty, war conflicts, drugs, diseases, security, terrorism, nuclear proliferation, and human rights issues, and it has suffered disappointing setbacks or complete failures in recent decades.

To improve sanitation and drinking water, the UN organizations FAO and OIE, and the WHO have assumed joint responsibility for addressing zoonotic and other diseases of potentially high socio-economic impact. These international UN organizations developed a Tripartite Concept Note (One Health) setting a course of action and proposing a long term framework for global partnerships which is oriented towards the coordination of global activities addressing health hazards and risks at the human-animal-ecosystems crossroads [122].

The One Health European Joint Program (OHEJP) is a European Commission co-funded scientific collaborative research program intended to help prevent and control food-borne and environmental contaminants that affect human health through joint actions on foodborne zoonoses, antimicrobial resistance, and emerging microbiological hazards [123]. 

Recognizing the health hazards and risks at the human–animal–ecosystems interface is a key element of their assessments, communication, and management. The One Health approach is considered critical for the emergence of antimicrobial drug resistance and on attending prevalent public health concerns, which comprise emerging infectious, parasitic, and zoonotic diseases [124]. Some 60% of human infectious diseases are of animal origin (zoonoses can be caused by bacteria, fungi, mycobacteria, parasites, viruses, and prions); nearly 75% of emerging human infectious diseases in the past three decades originated in animal-borne (even aquatic) diseases/pathogens [125]. Some 80% of such agents can be used for potential bioterrorism and are also pathogens of animal origin [126].

Through strong partnerships with human, animal, environmental health and civil society organizations and professionals, it is considered possible to stimulate advances concerning a safe and secure world with fewer infectious disease threats to human security. But while this UN One Health initiative has proven to be successful from an emerging and infectious disease perspective, its value still needs to be proven in terms of the exchanges and interactions of different microbiomes and elements of microbial communities’ transfer among humans, animals, and the environment [127].

## 6. One Health, Ecosystems and Veterinary Sciences

The development of new technologies to perform DNA sequencing has expanded studies on entire microbial communities in humans, animals, and in the environment. The term “microbiota” encompasses the entire complex ecosystem of gut microorganisms, the bulk of which reside mainly in the colon. The terms “microbiome” or the metagenome of the microbiota comprise all of the genetic material within a microbiota.

A complete understanding of human microbiomes in various body mucosa and surfaces requires an evolutionary perspective. The coevolution of humans and microbiota has generated host-specific microbiome structures and gut homeostasis of physiologic, metabolic, and antigenic diversity [128]. 

Population growth and economic development are leading to rapid changes in our global ecosystems [129]. Health risks are also a result of broader pressure on ecosystems, from the depletion and degradation of freshwater resources to the impacts of global climate change on natural disasters and agricultural production [130]. There is increased connectivity between humans, domestic pets, wildlife, farm animals, and real-world issues such as sanitation, economics, and food security. Ecosystems, landscapes, and a One Health paradigm, including social-ecological holistic approaches become increasingly important [131,132]. Such interactions require the integration of health science disciplines that span the spectrum from personalized care to public health [133].

At the national and international levels, these domains are organized in different Ministries, and there is an obstruction by professional corporatism which may impair the implementation of the One Health approach by not pursuing to unify health-related research. Furthermore, overcoming long-standing barriers of privacy and distrust among health professionals and political will are necessary to enable the integration of different health systems [134]. 

Fermentation, as a human ecological process, begins with the symbiotic human relationship with the microbial habitat [135]. Lifestyle, well-being, and even the survival of humans has been connected to single-celled microorganisms, i.e., fungi (yeasts) and bacteria on fermentation ecosystems [136]. The concept of a whole ecosystem is unpopular, and many have abandoned the idea that ecosystems have boundaries [137].

Gut microbes are extensively purged every one to two days and have the ability to double in number within the space of an hour [138]. In future the ecology of human nutrition may be studied on fermentation ecosystems models [139].

The One Health approach has been criticized for an excessive focus on emerging zoonotic diseases, inadequate incorporation of environmental concepts and expertise, and insufficient incorporation of social science and behavioral aspects of health and governance [140]. Barriers to implementing this strategy include competition over budgets, poor communication, and the need for improved technology [141]. 

At the national level, it is common to observe Veterinary Medicine, Animal and Veterinary Science, Colleges of Veterinary Medicine, and Veterinary Public Health within the Ministry of Agriculture and not in the Ministry of Health; therefore the link between animal health and human health is very precarious. Veterinary medicine is considered an Agrarian profession which does not include the concerns of Health professionals on most criteria, including resources. Environmental health is under the Ministry of Environment, and overall, this partition of responsibilities results in practical difficulties in terms of implementing the collaboration of multiple disciplines and sectors working locally, nationally, and globally to attain optimal health for people, animals, and the environment. 

At the international level, agencies such as The European Centre for Disease Prevention and Control (ECDC), The European Food Safety Authority (EFSA), the *forum* One Health European Joint Programme (OHEJP), and others, follow the developments on zoonoses with the mission of identifying, assessing and communicating current and emerging threats to human health posed by these diseases, as well as zoonotic agents, antimicrobial resistance, and food-borne outbreaks. However, the monitoring and surveillance schemes of most zoonotic agents are not harmonized between Member States, adding to existing complexity.

## 7. Concluding Remarks

Fermented food microbiology is an excellent model that is deeply connected to the dynamics that shape the human microbiota in different body sites. Perceiving microbial community interactions, essential for the threat of global antimicrobial resistance, will help to reveal, via a holistic approach, the unknown secrets of the human microbiome and the interactions which greatly influence multiple forms of human health, nutrition, well-being.

The relevance and potential of fermented foods and beverages, with contrasting and inconclusive results, and advocacy for their inclusion into dietary guidelines, depend on future clinical research. The limitations and inconsistencies in the current body of evidence mean that, presently, no definitive conclusions can be drawn on the potential health benefits of fermented products.

It is not easy to apply trans-inter-multi-disciplinary research required by the One Health approach due to its complexity, but the associated human-animal-environmental microbiota and health threats and risks demand that many challenges and handicaps must be overcome. After two decades, One Health still needs to prove its use and its ability to be applicable in parallel with the present bold reforms which are underway among major United Nations departments in order to more effectively respond to global crises, streamline activities, increase accountability, and ensure effectiveness.
